# Genotype and expression analysis of two inbred mouse strains and two derived congenic strains suggest that most gene expression is *trans *regulated and sensitive to genetic background

**DOI:** 10.1186/1471-2164-11-361

**Published:** 2010-06-07

**Authors:** Harry A Noyes, Morris Agaba, Susan Anderson, Alan L Archibald, Andy Brass, John Gibson, Laurence Hall, Helen Hulme, Sung Jong Oh, Stephen Kemp

**Affiliations:** 1 School of Biological Sciences, University of Liverpool, Liverpool, UK; 2 Biotechnology Theme, International Livestock Research Institute, Nairobi, Kenya; 3 National Institute of Animal Science, Rural Development Administration, Suwon, Korea; 4 The Roslin Institute and Royal (Dick) School of Veterinary Studies, University of Edinburgh, Roslin, Scotland, UK; 5 School of Computer Science/Faculty of Life Sciences, University of Manchester, Manchester, UK; 6 School of Science and Technology, University of New England, Armidale, New South Wales, Australia

## Abstract

**Background:**

Differences in gene expression may be caused by nearby DNA polymorphisms (*cis *regulation) or by interactions of gene control regions with polymorphic transcription factors (*trans *regulation). *Trans *acting loci are much harder to detect than *cis *acting loci and their effects are much more sensitive to genetic background.

**Results:**

To quantify *cis *and *trans *regulation we correlated haplotype data with gene expression in two inbred mouse strains and two derived congenic lines. Upstream haplotype differences between the parental strains suggested that 30-43% of differentially expressed genes were differentially expressed because of *cis *haplotype differences. These *cis *regulated genes displayed consistent and relatively tissue-independent differential expression. We independently estimated from the congenic mice that 71-85% of genes were *trans *regulated. *Cis *regulated genes were associated with low p values (p < 0.005) for differential expression, whereas *trans *regulated genes were associated with values 0.005 < p < 0.05. The genes differentially expressed between congenics and controls were not a subset of those that were differentially expressed between the founder lines, showing that these were dependent on genetic background. For example, the cholesterol synthesis pathway was strongly differentially expressed in the congenic mice by indirect *trans *regulation but this was not observable in the parental mice.

**Conclusions:**

The evidence that most gene regulation is *trans *and strongly influenced by genetic background, suggests that pathways that are modified by an allelic variant, may only exhibit differential expression in the specific genetic backgrounds in which they were identified. This has significant implications for the interpretation of any QTL mapping study.

## Background

There is considerable interest in discovering polymorphic loci that regulate differences in gene expression since this information can help us understand not only how genes are regulated but also how they interact. Genetic polymorphisms may regulate genes that are physically close to them on the chromosome (*cis *regulation) or anywhere else in the genome (*trans *regulation). There is a trivial sense in which all genes are expected to be both *trans *regulated by multiple transcription factors and *cis *regulated by transcription factor binding sites and this has been confirmed experimentally in yeast [1]. However the distinction between *cis *and *trans *regulation is only useful when the gene is differentially expressed between two conditions; then we can ask whether that difference is due to differences in the transcription factor binding region (*cis *regulated) or due to differences in structure or abundance of transcription factors (*trans *regulated) or both.

One strategy for the discovery of *cis *and *trans *acting genes is to use gene expression as a phenotype and to map associations between thousands of markers and the expression of thousands of genes in hundreds of samples to discover expression quantative trait loci (eQTL) [2,3]. This strategy routinely detects hundreds of *cis *regulatory loci since it is only necessary to test a few markers around each gene, but its statistical power to detect *trans *regulatory loci is limited by the large multiple testing correction that is required when correlating the genotype of thousands of genome wide markers with the expression of thousands of genes. The proportions of *trans *regulated genes discovered in these studies vary widely depending on the significance threshold used. Furthermore it has been argued that polymorphisms in transcription factors that cause extensive phenotypic effects are likely to be rare and rapidly purged from populations [4,5]. Consequently, although polymorphic network hubs that appear to regulate large numbers of genes have been detected, their discovery is very sensitive to the analysis strategy and probability thresholds used, they have rarely been confirmed experimentally and many may be false positives [4,6]. Therefore most *trans *regulators that effect multiple genes might have small individual effects making them hard to detect by genetic mapping and give the misleading impression that *cis *regulation is the dominant form. These problems make eQTL studies an unsatisfactory platform for discovering the relative contributions of *cis *and *trans *regulation to the observed differences in gene expression.

The availability of data for 8 million single nucleotide polymorphisms (SNP) in inbred mouse strains has been used to construct a comprehensive haplotype map of the mouse genome [7]. This makes it possible to determine the extent to which differences in gene expression are associated with haplotype differences. However since only 15 strains were genotyped there is insufficient statistical power to approach this question directly. Instead we have divided the list of ratios of gene expression between two mouse strains into one hundred groups with increasing p values for differential expression and then for each group asked whether there is a significant excess of genes with haplotype differences. This strategy makes it possible to identify the p values associated with differences in expression and haplotype and hence the contribution of *cis *effects to differential expression. Importantly this strategy also makes it possible to objectively identify a threshold p value at which there is a significant association between haplotype differences and gene expression. This value can then be used to quantify the contribution of *cis *regulation to differential regulation.

We estimated the contribution of *trans *regulation independently using expression data from a panel of congenic mice and their controls that had been developed for other purposes. Since congenic mice have a small region of donor genome introgressed into the host genome, any differential expression that is outside the introgressed congenic region can be assumed to be *trans *regulated by that region, permitting a direct estimate of the proportion of *trans *regulated genes. These *trans *regulated genes were highly enriched for cholesterol synthesis genes. This suggested that the congenic region was altering cholesterol flux and this in turn was causing changes in cholesterol gene transcription a process we call indirect *trans *regulation. Indirect *trans *regulation is likely to be even more sensitive to environmental and genetic effects than direct *trans *regulation by a transcription factor. It is therefore important to be aware of the extent of this effect.

## Results

Gene expression data were obtained for the parental and congenic lines for a total of 12 conditions (Table 1). Genes were defined as differentially expressed between lines if they had an absolute log_2 _difference in expression > 0.5 and a *pplr *less than the value indicated in the text (*pplr *is a measurement of probability that has the same characteristics as a p value, see materials and methods). The expression ratio and *pplr *for all relevant comparisons, together with the number of upstream SNP and the assigned haplotype is shown in Additional File 1 Expression+SNP+Haplotype.

**Table 1:** Microarray hybridisation conditions

Comparison	Tissues	No. arrays
C57BL/6 v A/J	Liver, Kidney, Spleen	5/5,5/5,5/5

Tir1CC v Tir1AA	Liver, Spleen	4/4,4/4

Tir3CC v Tir3AA	Liver	4/3

Conditions for which expression data was obtained. Expression was compared between three pairs of strains: C57BL/6 v AJ (parental strains); Tir1CC v Tir1AA and Tir3CC v Tir3AA. Pairs of values separated by a slash are the number of arrays hybridised for each of the pair of lines being tested. Values separated by commas are for the different tissues. The Tir*n*CC v Tir*n*AA pairs were lines derived from the homozygous littermates of an intercross between N7 heterozygotes for the congenic region (Tir*n*CA animals) that acted as controls for each other. Hence Tir1CC and Tir1AA mice are carriers of C57BL/6 and A/J alleles respectively at the *Tir1 *locus.

### Effect of sequence polymorphism on the probability of differential expression between inbred lines

Any SNP that causes a mismatch between probe and target might have a direct effect on the measure of expression by reducing signal and hence confound the observations [19], however Affymetrix arrays are probably relatively insensitive to this effect [6]. Since probes were designed against the C57BL/6 sequence any effect of mismatches on signal would be expected to lead to a loss of signal from A/J and an excess of genes that appear to be over-expressed in C57BL/6. The positions of each Affymetrix 25 mer probe in probesets that were scored as differentially expressed between A/J and C57BL/6 liver were retrieved from the Ensembl39 (NCBI36) database and compared with the position of SNP in the Perlegen (NCBI36) database. 59 out of the 797 differentially genes had SNP under probes but there was no evidence for an excess of SNP associated with probesets that were under-expressed in A/J (χ^2 ^0.21, 1 df, p = 0.64). Furthermore 49% more genes were over-expressed in A/J than C57BL/6; the opposite direction to any effect that would be expected to be caused by SNP modifying expression. Consequently the data could be used with confidence that observations were not a simple consequence of SNP causing expression differences (Additional File 2 SNP_under_Probes).

### Association between upstream SNP and differential expression

**Figure 1:**
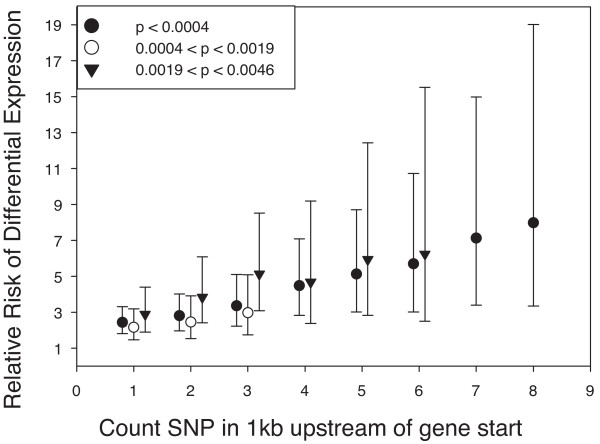
**Relative risk of differential expression for genes with *pplr *< 0.0046**. The relative risk that a gene would be differentially expressed given that it had *n *or more informative SNP in its 1 kb upstream region is shown for genes within the first three percentiles of the *pplr *distribution (*pplr *< 0.0046). The points on the graph have been spread around the integer values to make the individual error bars visible. There was no significant effect on relative risk for genes that had a *pplr *> 0.0046 after a Bonferroni correction for multiple testing. The error bars represent the 95% confidence interval of the relative risk.

Our objective was to identify any association between the number of upstream SNP and the probability of genes being differentially expressed. The genes represented in the liver data were ranked by *pplr *and divided into 100 groups of 193 genes each, representing the percentiles of the *pplr *distribution. For each percentile group the number of genes with each number of SNP between 1 and 10 in the 1 kb upstream region was obtained. For each percentile the number of genes with at least 1 upstream SNP was compared with the number with no upstream SNP by a χ^2 ^test. Then this was repeated for each number of upstream SNP between 2 and 10 Additional File 3 SNP_and_Expression). After a Bonferroni correction for multiple testing, there was only a significant association between upstream SNP and differential expression for the first three (most significant) percentiles of the *pplr *distribution, containing 580 genes with *pplr *< 0.0046, of which 246 were on different haplotypes. The relative risk of a gene being differentially expressed was obtained for genes with each number of SNP (Fig. 1). There was a strong correlation between the relative risk and the number of SNP in the upstream region for the first percentile (r = 0.99) and a similar relationship was observed for the next two but not subsequent percentiles.

The χ^2 ^tests showed highly significant associations with the presence of SNP and differential expression when *pplr *< 0.005. However there was no evidence for an association between the presence of SNP and genes that had *pplr *> 0.005. This was interpreted as evidence that the genes that are differentially expressed under the immediate control of upstream SNP have highly reproducible expression differences and hence give rise to low *pplr *values. In order to test this hypothesis the proportion of genes that were differentially expressed between the Tir1CC and Tir1AA mice and inside or outside the *Tir1 *congenic interval were compared for genes with *pplr *< 0.005 and 0.005 <*pplr *< 0.05. Of the 112 genes that were differentially expressed in *Tir1 *spleen or liver at the *pplr *< 0.005 confidence level, 23 were in the *Tir1 *congenic interval whereas of the 328 that were differentially expressed at the 0.005 <*pplr *< 0.05 confidence level only 7 were in the congenic interval (Fisher Exact Test; p < 10^-9^). This provided persuasive evidence that low *pplr *values are associated with haplotype differences.

### Estimating the frequency of differentially *cis *regulated genes between parental inbred mice

**Figure 2:**
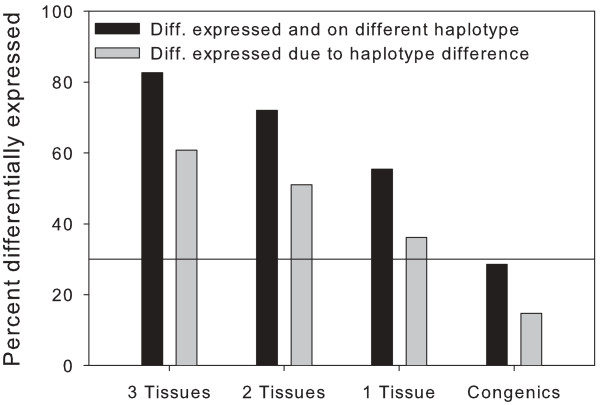
**Frequency of differentially expressed genes on different haplotypes and attributable to haplotype difference**. The proportion of the genes that were differentially expressed and also on different haplotypes in the parental C57BL/6 and A/J mice was obtained for all combinations of the three tissues at *pplr *thresholds of 0.005 (see Fig 3a for numbers). The percentages of genes that were differentially expressed and on different haplotypes are shown by the solid black bars, the percentages of genes that were estimated using Bayes Theorem to be differentially expressed due to haplotype differences are shown by the grey bars. 30% of the genes in A/J mice were on different haplotypes from C57BL/6, indicated by the horizontal bar on the plot. There was a significant excess of differentially expressed genes on different haplotypes in the parental mice but not in the congenic mice when the congenic region was excluded. Key "3 tissues" (kidney, liver, spleen) in parental mice (C57BL/6 and A/J); "2 tissues" - mean of the three combinations of two out of three tissues from the parental Mice; "1 tissue" - mean of individual tissues from the parental mice; "Congenics" - mean of the genes that were differentially expressed in the congenic mice and on different haplotypes in the parents for for the *Tir1 *(liver and spleen) and for *Tir3 *(liver) congenic excluding the congenic region. The percentage of genes that are differentially expressed in the congenic mice and on different haplotypes in the parents is expected to approximate to the percentage of genes on different haplotypes in the parents as was found to be the case.

The haplotype of the 1 kb upstream region differed between C57BL/6 and A/J for 30% of all genes for which haplotype data were available. This value is likely to be an estimate of the proportion of genes regulating any phenotype that can be discovered in a cross between these two strains. The extent to which genes are *cis *regulated may be reflected by the excess of those genes that are both differentially expressed and on different haplotypes over the number that would be expected to be on different haplotypes by chance. In the parental mice 30% of all genes were on different haplotypes in the two strains, but 55% of genes that were differentially expressed in at least one tissue were also on different haplotypes (χ^2 ^417, 1 df p < 10^-93^). Applying Bayes theorem shows that approximately 36% of genes (30%, 36% and 43% for spleen, kidney and liver respectively) that are differentially expressed in any one tissue can be attributed to haplotype difference and hence putatively *cis*-regulated (Fig. 2 and Additional File 4 Expression_and_Haplotype). 83% of genes that were differentially expressed in all three tissues tested in the parental strains had different haplotypes for the 1 kb upstream region and 61% of these genes may be differentially expressed due to the haplotype differences (Fig. 2). 51% of genes that were differentially expressed in two tissues were predicted to be due to difference in haplotype.

Genes that were potentially *cis *regulated, i.e. those with differential expression and on different haplotypes, were also five times more likely to be differentially expressed in multiple tissues than genes that are potentially *trans *regulated: 46/268 (17%) of genes that were differentially expressed in at least one tissue and on different haplotypes (putatively *cis *regulated) were differentially expressed in all three tissues, whereas only 7/214 (3%) of genes that were differentially expressed in at least one tissue and on the same haplotype (putatively *trans *regulated), were differentially expressed in all three tissues (Fisher exact test, p = 10^-7^). This suggests that *cis *effects were five times less sensitive to tissue environment than *trans *regulated genes.

### Effect of genetic background on differential expression

**Figure 3:**
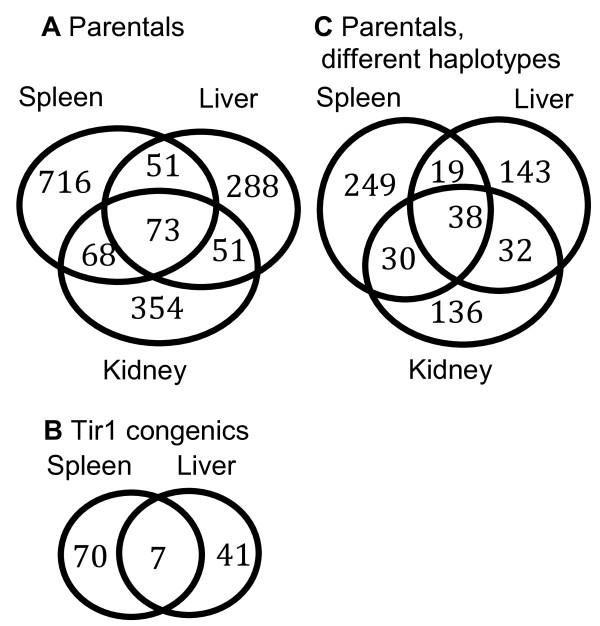
**Venn diagrams, showing the numbers of genes differentially expressed in each tissue**. **A **Parental A/J vs C57Bl/6, **B **Congenic Tir1AA vs Tir1CC, **C **genes in the parental lines which occur on different haplotypes and are differentially expressed. In all cases most genes were differentially expressed in only one of the tissues. Differential expression was defined as absolute log_2 _fold change > 0.5 and *pplr *< 0.005.

The number of genes that were differentially expressed in each condition or in relevant combinations of conditions are summarised in figure 3. As expected, there was little overlap in the lists of genes differentially expressed in different tissues. In the parental lines for example (Fig. 3a), 85% of all differentially expressed genes are differentially expressed in only one of the three tissues.

**Figure 4:**
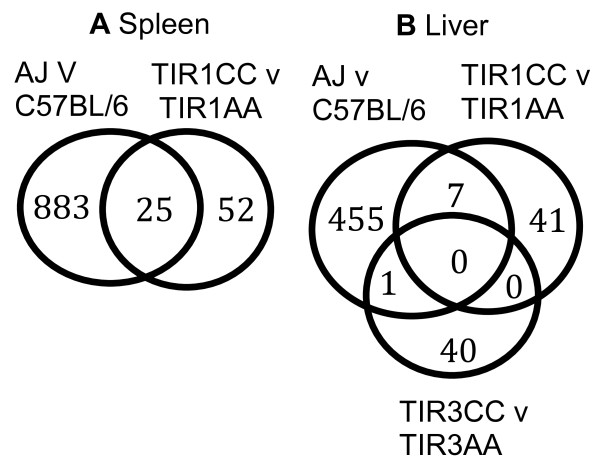
**Venn diagrams, showing the numbers of genes differentially expressed in each condition**. **A **spleen, **B **liver. Only 3 of the 25 genes differentially expressed in both parental and congenic spleen were outside the congenic interval and none of the 7 genes that were differentially expressed in the liver in both parental and *Tir1 *congenic mice were outside the congenic interval. Differential expression was defined as absolute log_2 _fold change > 0.5 and *pplr *< 0.005.

Surprisingly however, there was relatively little overlap between the sets of genes that were differentially expressed in the congenic strains and in the parental strains from which they were derived (Fig. 4). In the spleen only 3/77 genes that were differentially expressed in the congenic mice were also differentially expressed in the parents and outside the congenic interval. In the liver there were no genes that were outside the congenic interval and differentially expressed in both congenics and parents. This is less than would be expected by chance in each case and shows that the *trans *effect of genes within the congenic interval on genes outside the interval is not detectable in the parental mice. Microarray studies to detect pathways that cause the difference in phenotype between strains are based on the assumption that the difference in expression of those pathways will be substantial enough to be observable. In this case there was no observable difference in the parental strains in any of the pathways that were regulated by the QTL in the congenics and therefore expression data from the parental strains may not be a reliable resource for identifying the pathways that regulate the phenotype.

### Estimating the proportion of *trans *regulated genes

**Figure 5:**
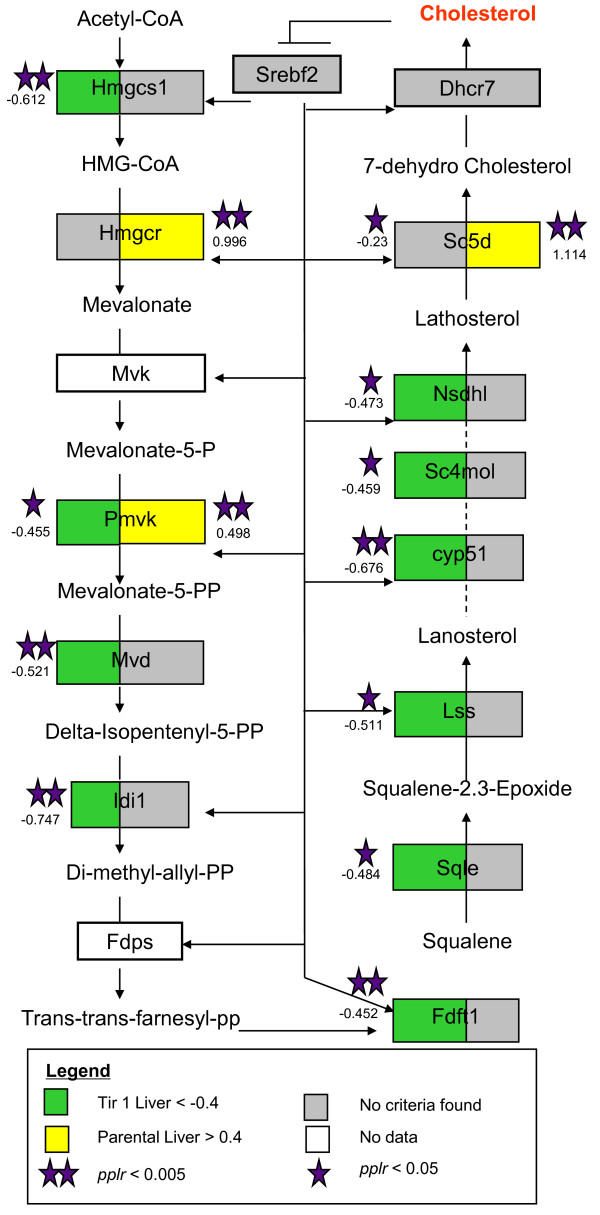
**Cholesterol synthesis pathway compiled using GenMapp**. Genes regulated by *Srebf2 *(*Srebp2*) from Reed et al. [33]. Ratio of Tir1CC to Tir1AA congenic mouse expression is shown on the left of each box and the ratio of C57BL/6 to A/J parental mouse expression is shown on the right of each box. Genes in green boxes have < -0.4 log_2 _expression ratio in the liver of the congenic mice, genes in yellow have > 0.4 log_2 _expression ratio in the liver of parental mice. Genes indicated by purple stars are significantly differentially expressed as indicated in the legend. Log_2 _expression ratios are shown by each gene where the absolute ratio > 0.4 or the ratio is significantly different from 0 as indicated by the stars. Negative log_2 _ratios indicate higher expression in mice carrying A/J alleles (A/J and Tir1AA) than mice carrying C57BL/6 alleles (C57BL/6 and Tir1CC). None of the genes in this pathway were differentially expressed in Tir3AA v Tir3CC.

In the spleen of the congenic mice 55 of 77 (71%) differentially expressed genes were outside the congenic interval and therefore putatively *trans *regulated. In the congenic liver (Fig. 4b) 41/48 (85%) of genes appeared to be *trans *regulated. The most prominent group of *trans *regulated genes was the cholesterol and steroid synthesis pathway, which was significantly differentially regulated (p = 2.55^-7^) in the liver between Tir1CC and Tir1AA mice but no difference in this pathway was observed between the parental or the Tir3NN strains (Additional File 5 GO_KEGG). Eleven of the thirteen genes in the steroid biosynthesis pathway between *Hmgcs1 *and *Dhcr7 *were significantly over-expressed in the liver of Tir1AA mice *pplr *< 0.05 (Fig. 5). In contrast only 3 out of the thirteen had significantly higher expression in parental C57BL/6 than in A/J, the opposite direction of effect and not enough differentially expressed genes to cause the pathway to be flagged as differentially expressed.

## Discussion

The availability of high-density genotype data for mice has made it possible to quantify the relationship between haplotype structure and differential gene expression. A relationship between SNP in the 1 kb upstream region and differential expression was only observable when using a stringent test of differential expression (absolute log_2 _fold change > 0.5; *pplr *< 0.005). This was interpreted as evidence that the variance of expression of *cis *regulated genes is much lower than that of *trans *regulated genes. Although the 1 kb upstream region may contain the highest density of regulatory elements it does not contain all of them, they can be spread throughout the gene and its 3' region as well as going tens to hundreds of kilobases upstream. Therefore we are likely to have underestimated the numbers of *cis *regulated genes using this strategy. However SNP in the upstream region will frequently be markers for larger haplotypes that extend into or through the whole gene region so these regions will not have been completely excluded from the analysis. By selecting the region with the highest density of regulatory elements we have maximised our power to detect an association between SNP and gene expression, which would have been more difficult in the presence of higher proportions of non-functional SNP distributed through the rest of the gene region.

If SNP within probe positions were directly affecting signal then this would confound our results. Since the probes were designed against C57BL/6 sequence any SNP that effected signal would be expected to reduce signal in A/J relative to C57BL/6. However we have shown that only 59 of the 797 differentially expressed genes in the liver had SNP under probes and even for these 59 there was no association between their presence and reduced signal in A/J. Therefore we do not consider that SNP under probes is a source of bias in our data.

By examining the correlation between gene expression differences and haplotype allele differences in A/J and C57B/6 mice we estimated that haplotype differences accounted for 36% of expression differences and these were assumed to be *cis *regulated, although as noted above this is likely to be an underestimate. We independently estimated that 71 and 85% of genes were outside the congenic interval and hence *trans *regulated in the spleen and liver of the *Tir1 *congenic mice respectively. This also likely to be an underestimate since some genes within the congenic interval may also be *trans *regulated. The combined data suggests that there might be an approximately 2:1 ratio of *trans*:*cis *regulated differences in gene expression. Clearly this is a preliminary estimate based on a very small sample but it emphasises the dominance of *trans *regulation.

Genes are both *cis *and *trans *regulated and by multiple *cis *binding sites and multiple *trans *acting factors. However the 2:1 ratio may reflect the contributions of *trans *and *cis *factors to the observed differences in expression. It is well established that there are large differences in gene expression between tissues [20] and this observation was replicated here. Indeed this must be so for cell types to differentiate, thus underlining the sensitivity of gene expression to environment and this regulation must be by a *trans *mechanism. A study of mouse brain regions in multiple mouse strains found that region specific transcription was mainly *trans *regulated whilst strain specificity was mainly *cis *regulated [20].

The cholesterol synthesis pathway appeared to be *trans *regulated by the *Tir1 *region in the congenic mice but this was not observable in the parental mice. This pathway is regulated by the transcription factor SREBF2, which is inactive when cholesterol concentrations are high but migrates to the nucleus when cholesterol is low to bind transcription factor binding sites in cholesterol pathway genes and promote transcription; thus forming a negative feedback loop on cholesterol synthesis [21]. The co-ordinated up-regulation of the cholesterol pathway in Tir1AA mice implies that SREBF2 is responding to lower cholesterol levels in the livers of Tir1AA mice than Tir1CC mice. Since cholesterol synthesis is co-ordinately regulated by an end-product feedback mechanism through SREBF2, the most likely explanation for the co-ordinated response is that some gene or genes in the *Tir1 *congenic region is modifying cholesterol flux (but not necessarily serum cholesterol levels) by changing the rate of absorption or excretion from cells or the body. There are at least two genes in the *Tir1 *region that are directly involved in cholesterol metabolism (*Abcg1 *which participates in cholesterol efflux from the cell and *Rxrb *which regulates *Abc *mediated efflux[22]) but cholesterol plays an important role in the response to infection and *Tnfa *which is also in the *Tir1 *region has been shown to regulate cholesterol as well [23,24], any of the other immune related genes in the MHC region could also be contributing to changes in cholesterol flux. We cannot exclude the possibility that a novel transcription factor in the *Tir1 *region may be competing with SREBF1 to directly regulate cholesterol pathway transcription. However, since there is no evidence in our data to suggest that a novel mechanism is responsible for our observations, it is most parsimonious to assume that the standard model holds here and the cholesterol pathway is responding to changes in cholesterol flux through the liver. Since no significant difference in cholesterol pathway expression was observed in the parental strains we assume that multiple other interacting processes are buffering the effect of the *Tir1 *region in these lines, as would be expected if the region was acting by modifying cholesterol flux rather than acting through a transcription factor.

We describe this mechanism of regulation by multiple intermediates such as metabolites as *indirect trans *regulation to distinguish it from *direct trans *regulation of a target gene by a transcription factor. Cholesterol synthesis is a particularly well-known example of *indirect trans *regulation [25].

Almost completely different sets of genes were found to be differentially expressed between the congenic mice and their controls and between the parental inbred mice, as has been observed before [26]. This suggests that the problem of identification of *trans *regulated genes goes well beyond the lack of statistical power of eQTL studies for this purpose. Expression differences in *cis *regulated genes were found to have lower variances than those of *trans *regulated genes in this study, and several eQTL studies have found that *cis *regulated differences in transcription are larger than *trans *regulated differences [3]. This is to be expected since *trans *regulated genes are likely to have a much larger number of intermediates involved in their effect on expression. Firstly there is usually a complex of gene products and metabolites that bind to a given regulatory factor binding site, eg a mean of 3.1 loci have been found to regulate each *trans *regulated gene in radiation hybrid cells [27]. Secondly the concentrations and activities of members of that complex may be regulated by other molecules elsewhere that regulate translocation to the nucleus. Consequently the regulatory binding site acts as a transponder for the whole cascade of events leading to regulatory complex binding or activation, for example a study in yeast found that *trans *acting eQTL were not enriched for transcription factors and that a wide range of gene classes could cause *trans *differences in expression [1]. This suggested that most differences in gene expression were caused by *indirect *rather than *direct *(transcription factor mediated) *trans *effects. Each interaction in the regulatory cascade is likely to introduce an element of noise and hence the effect of a *trans *acting regulatory factor polymorphism on gene expression is likely to have much higher variance than a *cis *polymorphism. Therefore *indirect trans *regulation is likely to have even larger variance than *direct trans *regulation. These higher variances will make *trans *regulated differences in expression between conditions much harder to detect than *cis *regulated differences and *indirect trans *harder than *direct trans*. We do not know the proportion of genes that were *indirectly trans *regulated in the congenic mice; there are several zinc finger proteins with unknown targets in the congenic region that might be *direct trans *regulators. *Indirect trans *regulation relationships are likely to be less well known because they are more complex and harder to discover experimentally by methods such as chromatin immunoprecipitation. But if the cholesterol synthesis pathway is representative of the main mode of *trans *regulation in the congenic mice, most regulation was *indirect*.

The greater sensitivity of *trans *regulated expression to experimental conditions than *cis *regulated genes may make it even harder to detect those genes that are subject to both polymorphic *trans *and *cis *factors. Since the penetrance of the *cis *acting factors is usually likely to be higher than the *trans *factors only the strongest *trans *effects will be observable in the presence of concurrent *cis *effects.

The large proportion of *trans *regulated genes is important because their regulation appeared to be less stable under different conditions and consequently gene expression based approaches to the discovery of genes regulating a phenotype are likely to be exquisitely sensitive to the particular experimental conditions used. There was almost no overlap between genes that were differentially expressed between congenics and controls and outside the congenic regions, and genes that were differentially expressed between the parental inbred mice strains, even when comparing the same tissue, and this is consistent with previous observations [26]. This indicates that the observable *trans *regulation of gene expression is highly context dependent; the context in this case being genetic background. A study of recombinant inbred strains of mice estimated that 1500 genes differed between conditions and 1200 were sensitive to genetic background and hence presumably *trans *regulated [28]. The sensitivity of phenotype to genetic background in which a trait is expressed has also been dramatically illustrated by a study of 41 selected traits in a complete panel of chromosome substitution strain mice (CSS) that found that for 56% of phenotypes the sum of the trait differences from the host strain over all CSS lines exceeded 500% of the difference between the host and donor strain [29].

The sensitivity of both expression and phenotype to genetic background has profound implications for the interpretation of expression data for a range of purposes. For example, the use of expression arrays is a common strategy for following-up QTL mapping studies with the objective of identifying the genetic polymorphism underlying a QTL. Gene expression is typically measured in the parental lines that are used to generate the mapping populations in order to infer the genes regulated by the QTL. The candidate quantative trait gene(s) (QTG) causing the phenotypic difference may be identified because they are differentially expressed in at least one tissue. Candidate genes can be selected from these long lists by identifying those that participate in networks that are differentially expressed and that intersect with the QTL region. We have previously identified a candidate gene (*Daxx*) in the *Tir1 *QTL region that is not differentially expressed, but was subsequently found to have an amino acid indel, on the basis of its membership of a differentially expressed KEGG pathway in the parental strains [30]. However it is clear from the data presented here that there is no reason to expect that the genes and pathways that are regulated by the QTL gene(s) will respond in the same way in the parental mice as they will in the mapping populations and hence the pathways that differ between the extremes of the mapping population may not be detected by measuring gene expression in the parental mice.

The observation that the gene expression phenotype of congenic mice is overwhelmingly not predictable from the expression of genes in the parental strains has consequences for both QTL discovery and exploitation. It begs the question of why congenic mouse lines often retain the expected trait associated with the introgressed QTL. The explanation may be that most QTL that are discovered in mapping populations are those that are caused by alleles that are insensitive to genetic background. This would explain why mapping studies that have used parental strains with limited genetic differences have discovered unusually large numbers of QTL [31]. This could mean that conventional QTL mapping approaches fail to map the bulk of the theoretical potential of any given quantitative trait, not because the unmapped fraction is associated with multiple loci of small effect but because it is associated with loci that may be of large effect only on particular genetic backgrounds. If this is the case then the large number of inbred mouse strains being generated by the collaborative mouse cross [32] are likely to reveal far more loci regulating phenotypic differences than a simple comparison between the eight founder lines would predict.

## Conclusions

We have found that *cis *regulated genes are associated with low *pplr *values (<0.005), presumably because the close coupling of the polymorphic regulatory region to the gene leads to much lower variances in expression. There was a ratio of approximately 2:1 of *trans:cis *regulated genes and the *cis *regulated genes were more likely to be differentially expressed in multiple tissues than *trans *regulated genes. The fact that genes that are regulated by a congenic region were not observably regulated in the parental mice, means that expression studies in the parents of a mapping population are unlikely to detect many of the *trans *regulated expression differences caused by the QTL genes. This will make it much harder to identify the pathways regulated by a QTL. The *Tir1 *region of chromosome 17 regulated the cholesterol synthesis pathway, but this was probably because this region modifies cholesterol flux and not because it contains transcription factors that regulate cholesterol metabolism. This mode of *indirect trans *regulation may be the most common form of gene regulation but also the hardest to detect except in well defined genetic backgrounds such as congenic mice. Consequently only the *cis *and largest *direct trans *regulatory relationships will be amenable to discovery and the large fraction of *indirect trans *regulation will be missed by most high throughput experimental strategies.

## Methods

### Congenic mice

All mouse work was conducted at the International Livestock Research Institute in Nairobi and approved by their Internal Animal Care and Use Committee. In the UK no project licence would be required for the procedures described here since they were all conducted on *post mortem *animals that had not been subject to any prior treatment.

Two congenic lines were created to cover the *Tir1 *and *Tir3 *QTL for *Trypanosoma *infection response [8,9] as previously described [10]. Two congenic lines were created corresponding to predicted *Trypanosoma *infection response (*Tir*) loci; *Tir1 *and *Tir3 *on chromosomes 17 and 1, respectively. The progeny at each backcross generation were genotyped with microsatellite markers defining the genetic intervals containing the QTL using the following markers: D17Mit29, D17Mit16 and D17Mit11 (*Tir1*); D1Mit60, D1Mit217 and D1Mit87 (*Tir3*). At the seventh generation of backcrossing each line was typed with a series of markers at approximately 2 cM intervals flanking the *Tir *loci. The individuals with the shortest donor haplotype extending beyond the QTL interval were used for breeding the next generation by intercrossing a single heterozygous male with full or half sib female carriers of the C57BL/6 donor region. The progeny of these were genotyped and those individuals homozygous for the alternative haplotypes were used as founders to propagate each line, which were denoted either TirnAA or TirnCC for homozygotes at the QTL for recipient A/J haplotype and the donor C57BL/6 haplotype, respectively. Thus in total 4 lines were produced; these are Tir1AA and Tir1CC; Tir3AA and Tir3CC. The Tir1CC line is homozygous for a C57BL/6 haplotype spanning 10 cM interval between marker D17Mit84 and D17Mit177 on Mmu17. Tir3CC individuals have a C57BL/6 haplotype spanning approximately 10 cM between markers D1Mit49 and D1Mit139 on Mmu1.

The recommended names for the test lines according to the Mouse Genome Informatics would be A.B6-*Tir1 *and A.B6-*Tir3*. However there is no recommended nomenclature for the control lines, so for clarity the TirnAA and TirnCC style will be used in the following description.

The use of controls derived from the same line as the congenic mice is critical since even the small amount (<1%) of non target C57BL/6 remaining can have profound non-specific effects on phenotype through heterosis. The homozygous congenic mice lines were genotyped at the Wellcome Trust Clinical Research Facility, Edinburgh, UK using the Illumina Mouse Medium Density Linkage Panel on an Illumina BeadStation 500 instrument, with the Illumina 1536 murine SNP panel. 959 markers were informative between A/J and C57BL/6 with a mean spacing of 2.61 Mb. Using this data it was possible to identify the approximate boundaries of the introgressed regions and also to identify non-target regions of C57BL/6 origin that had also been carried through into the congenic lines.

This showed that Tir1CC congenic mice carried a region of C57BL/6 between 26.0-43.9 Mb (NCBI36) on Mmu17 in the A/J background, this region includes the major histocompatibility complex (MHC). The Tir3CC mice carried a region of C57BL/6 origin between 93.3-123.6 Mb on Mmu1. The SNP genotyping data showed that approximately 0.75% of the genome outside the congenic regions was of C57BL/6 origin in both TirNAA and TirNCC mice [10]. The non-target regions were not fixed in the mice that were genotyped and no differentially expressed genes were observed in these regions. It is impossible to exclude the possibility of *trans *effects from these loci, however since they were present but not fixed in both test and control mice we would have had very low power to detect *trans *effects from the non-target loci, therefore we have disregarded possible effects from these loci in the subsequent discussion.

The initial cross and the first three backcrosses were reciprocal between the sexes, the fourth backcross was of congenic females to A/J males to eliminate residual C57BL/6 Y chromosomes. Subsequent backcrosses were of a single congenic male to multiple A/J females. The Y chromosome was not assayed in the SNP genotyping so the Y chromosome genotype has not been independently verified.

### Expression analysis

Affymetrix Mouse 430 2.0 microarrays were used for expression profiling of each of the two congenic lines, their respective controls and the parental A/J and C57BL/6 mice as part of a larger study of response of the transcriptome to infection with *Trypanosoma congolense *[11]. C57BL/6JOlaHSD (C57BL/6) and A/JOlaHsd (A/J) mice were purchased from Harlan UK housed at the ILRI facility on a twelve-hour light/dark cycle and fed mouse diet SDS-RM3 (E) (4.2% fat, 22.4% protein; Special Diets Services, Witham, Essex UK) and water *ad libitum *for 4 weeks to acclimatise before killing. Tissues for hybridisation were selected iteratively and different sets of tissues were hybridised for each pair of strains (Table 1). RNA from liver, spleen and kidney of the parental lines were hybridised first and the data showed that the liver, spleen and kidney varied in the differences that they exhibited between strains. The kidney differed least between parental strains and was excluded from the subsequent experiments on the congenic mice. The liver was found to give the most reproducible signals, with the most differentially expressed genes and be informative for many immune related functions and was hybridised for all lines. The differences in the liver between *Tir3 *test and control mice were very small and consequently spleen data was not collected for these animals.

Congenic mice were used at the second generation after being made homozygous. Congenic mice were co-housed under the same conditions as the parental mice and equal numbers of each line were simultaneously killed for tissue collection in three batches 1 year after the inbred mice at a mean age of 20 weeks. All batches of mice were killed at 2 pm to minimise diurnal variation. For each strain RNA was prepared from various tissues of twenty or twenty-five mice. Tissues were ground under liquid nitrogen and extracted with Trizol (Invitrogen) and further purified on RNAeasy columns (Qiagen). RNA was quantified and checked for integrity on an Agilent Bioanlyser and extractions were repeated for any degraded samples. The samples were then mixed into four or five pools of five samples for each strain, most pools comprised of samples of a single sex. The pooling strategy that we used is predicted to give the same power to detect differential expression as over fifteen individual samples hybridised separately [12]. Sex was balanced in each set. For the congenic mice we compared chips by sex to identify sex effects as well as by strain to identify strain effects and also mixtures of strain and sex to identify background noise. Twice as many genes were differentially expressed between the sexes as between the strains but there was no overlap between the two sets, ie no genes that were differentially expressed between the sexes were also differentially expressed between congenic test and control lines. Consequently we are confident that our observations are not due to sex effects.

Chips were initially assessed using DChip criteria and PCA to screen for outliers. Data from outlier chips were discarded and hybridisations were repeated with the same or fresh RNA samples from the same batch of mice until complete sets of chips for each condition were obtained. Sets of 25 mer probes for each gene represented on the array were identified using AffyProbeMiner [13]. Normalisation was carried out in the R environment using multi-mgmos [14] and differential expression was also assessed in R using PPLR (Probability of Positive Log-Ratio) [15]. The PPLR method uses Bayesian techniques to detect differentially expressed genes. The method assigns a confidence statistic, taking values between 0 and 1, based on the reproducibility of the probe-level measurements. A score of close to 0 or close to 1 indicates evidence of an exceptional event, namely up-regulated or down-regulated expression between conditions respectively. Common events have values around 0.5. In the analysis presented here we have used the minimum of (PPLR, 1- PPLR), which we denote *pplr *as a confidence value, for ease of comparison and ranking of genes such that a value close to zero indicates significant confidence in differential expression. PPLR makes use of the probe-level measurement error across replicated experiments and may be most appropriate where there are a variable number of probes per probeset, as is the case with probesets obtained from AffyProbeMiner. The data is available through ArrayExpress under accession numbers E-MEXP-1190 for the parental strains and E-TABM-865 for the congenic strains.

### Associations of SNP and Expression

The Perlegen SNP dataset [7] was downloaded into a MySQL database. A list of all genes in the genome together with their start positions and strand was similarly downloaded from Ensembl using the NCBI36 mouse genome assembly. For each Affymetrix probe set, the number of SNPs between C57BL/6 and A/J in the 1 kb upstream region of the gene to which the probe was targeted was obtained from the database. This information was used to obtain the counts of genes with absolute log_2 _fold difference between strains >0.5 and *pplr *values for a difference in expression below the thresholds indicated in the text.

### Associations of haplotype and expression

Boundaries of haplotypes identified within the Perlegen SNP data set were downloaded from the UCLA Perlegen Mouse SNP Browser Mouse[16], strains were allocated to haplotypes at each haplotype block using a local Perl script that extracted all alleles from the Perlegen dataset within a haplotype block, aligned them on the basis of genomic positions provided with the data and submitted them to the Jukes-Cantor algorithm in DNADIST in PHYLIP to calculate genetic distances between strains [17,18]. The distribution of distances was examined and a threshold of 0.2 was selected, such that strains within a distance of 0.2 were allocated to the same haplotype block using C57BL/6 as the reference strain (See Additional File 6 Haplotype block assignment). Haplotype assignments for each Haplotype block are shown in Additional File 7 Haplotype_block_alleles, 13,385 Ensembl genes could be assigned to haplotypes.

### Estimating the frequency of differentially *cis *regulated genes using Bayes theorem

Bayes theorem was used to estimate the proportion of differentially expressed genes that were putatively differentially expressed because of difference in haplotype.

Bayes Theorem: ((N_h_/E) × (N_hd_/N_h_))/( G_d_/E)) where N_h _is the number of differentially expressed genes for which haplotype data was available; N_hd _is the number of differentially expressed genes on different haplotypes; G_d _Number of genes for which C57BL/6 and A/J have different haplotypes; E is the Number of Ensembl genes with haplotype data. Values for each variable and condition are shown in Additional File 4 Expression_and_Haplotype.

### Identification of pathways in differentially expressed genes

Pathways that were overrepresented amongst the differentially expressed genes were identified using KEGG, DAVID and GeneGO and are shown in Additional File 5 GO_KEGG.

## Abbreviations

eQTL: expression quantative trait locus; KEGG: Kyoto Encyclopaedia of Genes and Genomes.

## Authors' contributions

HN did the SNP analysis and drafted the manuscript; MA managed experimental work on the mice; SA managed microarray work; AA participated in study design and supervised microarray work; AB analysed microarray data and interpreted the analyses; JG designed mouse breeding and supervised mouse work; LH did microarray hybridisations and quality control; HH analysed microarray data, SJO data interpretation, SJK conceived of the study, interpreted data, redrafted manuscript, supervised study. All authors read and approved the final manuscript.

## Supplementary Material

Additional file 1 **Expression ratios and *pplr *values for all conditions for all genes with numbers of upstream SNP and Haplotype block assignment for A/J**. Click here for file

Additional file 2 **Counts of SNP under each probeset and calculations of their effect on observed expression differences**. Click here for file

Additional file 3 **Numbers genes with each number of SNP in their upstream region and the correlation with expression differences**. Click here for file

Additional file 4 **Calculations of number of differentially expressed genes attributable to haplotype differences**. Click here for file

Additional file 5 **GO and KEGG classifications of differentially expressed genes**. Click here for file

Additional file 6 **Description of how allele numbers were assigned to haplotype blocks**. Click here for file

Additional file 7 **Allele assigned to each strain at each haplotype block**. Click here for file
