# Supplementary material for: Genotype and expression analysis of two inbred mouse strains and two derived congenic strains suggest that most gene expression is trans regulated and sensitive to genetic background
Source: BMC Genomics. 2010 Jun 7;11:361. doi: 10.1186/1471-2164-11-361 (PMC2896378; doi:10.1186/1471-2164-11-361)
Supplement: Additional file 6 — Description of how allele numbers were assigned to haplotype blocks. [file 1471-2164-11-361-S6.DOC]

Allocation of strains to haplotypes for each of the haplotype blocks identified by Frazer et al 2007.

Haplotype boundaries were downloaded from <http://mouse.cs.ucla.edu/perlegen/block_summary.txt> (Frazer et al. 2007). Strains were allocated to haplotypes for each haplotype block using a local script that extracted all alleles from the Perlegen dataset within a haplotype block, aligned them based on genomic positions provided with the data and submitted them to the Jukes-Cantor algorithm in DNADIST in PHYLIP to calculate genetic distances between each pair of strains (Felsenstein 2005). Strains were given a binary “barcode” with a all possible pairs of strains assigned a 1 or a 0 depending on whether the genetic distance for that pair was above or below a threshold value. Strains that had the same”barcode” were allocated to the same haplotype number. C57BL/6 was used as the reference strain for block allocation and assigned to haplotype one, succeeding strains were allocated to the same haplotype block as another strain they shared a haplotype with or if there was none to the next available haplotype number. See the example haplotype numbers in table 1 for an example of haplotype numbers with eight strains. Haplotype assignments for each Haplotype block used in the present study showing Jukes-Cantor distances, “barcodes” and haplotype numbers are shown in Supplementary_Data_File_2_Haplotype_Block_alleles.txt. 13385 Ensembl genes could be assigned to haplotypes. The script is available from the authors on request.

The distribution of distances between strains was examined in order to determine the most appropriate threshold value to use to allocate strain to the same or different haplotypes (Fig. 1).

Figure 1. Cumulative frequencies of mean distances between strains. PWD and CAST are evidently the most distantly related to the other strains as expected for these two wild derived strains.

There was no obvious inflection in the cumulative frequency plot at which to set a threshold distance for assigning blocks to the same or different haplotypes. Three different threshold distances (0.1, 0.2 and 0.3) were tested in order to determine the sensitivity of the distribution of haplotype frequencies to different threshold distances.


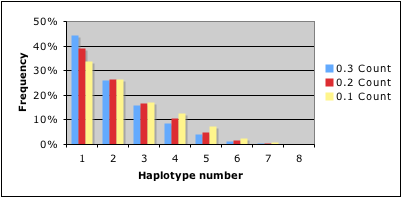
FiFigure 2 Distribution of haplotype numbers after using each of three threshold distances for the allocation of 8 strains to haplotypes.

Although the choice of distance for the cut off clearly has an effect on the distribution the effect is not substantial. A distance of 0.2 was arbitrarily chosen for the studies described in the main text.

| Haplotype block | Chromosome | start | end | C57BL6 | A_J | 129S1_SvImJ | NOD_LtJ | WSB_EiJ | PWD_PhJ | CAST_EiJ | NZW_LacJ |
| --- | --- | --- | --- | --- | --- | --- | --- | --- | --- | --- | --- |
| 1 | Chr01 | 6265 | 16271 | 1 | 1 | 2 | 2 | 3 | 4 | 5 | 2 |
| 2 | Chr01 | 36839 | 51355 | 1 | 1 | 1 | 2 | 2 | 3 | 4 | 1 |
| 3 | Chr01 | 3039187 | 3104448 | 1 | 1 | 2 | 2 | 3 | 4 | 5 | 1 |
| 4 | Chr01 | 3104580 | 3119136 | 1 | 1 | 2 | 3 | 4 | 5 | 2 | 1 |
| 5 | Chr01 | 3119380 | 3124939 | 1 | 1 | 2 | 2 | 1 | 3 | 4 | 1 |
| 6 | Chr01 | 3125312 | 3137037 | 1 | 1 | 2 | 2 | 1 | 3 | 4 | 1 |
| 7 | Chr01 | 3152711 | 3437350 | 1 | 1 | 2 | 2 | 3 | 4 | 5 | 1 |
| 8 | Chr01 | 3438875 | 3489313 | 1 | 1 | 2 | 2 | 2 | 3 | 4 | 1 |
| 10 | Chr01 | 3503693 | 3597668 | 1 | 1 | 1 | 1 | 1 | 2 | 3 | 1 |
| 11 | Chr01 | 3598904 | 3606142 | 1 | 1 | 1 | 1 | 1 | 2 | 3 | 1 |
| 12 | Chr01 | 3612766 | 3931231 | 1 | 1 | 1 | 1 | 2 | 3 | 4 | 1 |
| 13 | Chr01 | 3938137 | 3962477 | 1 | 2 | 3 | 3 | 4 | 5 | 6 | 2 |
| 14 | Chr01 | 3970351 | 3977423 | 1 | 2 | 1 | 1 | 3 | 4 | 5 | 1 |
| 15 | Chr01 | 3981463 | 4342023 | 1 | 1 | 1 | 1 | 2 | 3 | 4 | 1 |
| 16 | Chr01 | 4342313 | 4425482 | 1 | 1 | 1 | 1 | 2 | 3 | 4 | 1 |

Table 1 Example of haplotype numbers assigned to each strain in each block on the basis of distance calculated by DNADIST in PHYLIP C57BL/6 was used as the reference strain and haplotype numbers were assigned incrementally for each new haplotype. For this example a threshold distance of 0.1 was used as a cut off to allocate haplotype numbers to strains. Haplotype assignments for each Haplotype block used in the present study are shown in Supplementary_Data_File_2_Haplotype_Block_alleles.txt

Felsenstein, J. 2005. PHYLIP (Phylogeny Inference Package) version 3.6.

Frazer, K.A., E. Eskin, H.M. Kang, M.A. Bogue, D.A. Hinds, E.J. Beilharz, R.V. Gupta, J. Montgomery, M.M. Morenzoni, G.B. Nilsen, C.L. Pethiyagoda, L.L. Stuve, F.M. Johnson, M.J. Daly, C.M. Wade, and D.R. Cox. 2007. A sequence-based variation map of 8.27 million SNPs in inbred mouse strains. *Nature*.
